# Cusp Catastrophe Regression and Its Application in Public Health and Behavioral Research

**DOI:** 10.3390/ijerph14101220

**Published:** 2017-10-13

**Authors:** Ding-Geng Chen, Xinguang Chen

**Affiliations:** 1 School of Social Work, University of North Carolina, Chapel Hill, NC 27599, USA; 2 Department of Biostatistics, Gillings School of Global Health, University of North Carolina, Chapel Hill, NC 27599, USA; 3 Department of Epidemiology, College of Public Health & Health Professions, College of Medicine, University of Florida, Gainesville, FL 32610, USA; jimax.chen@ufl.edu

**Keywords:** cusp catastrophe regression, maximum likelihood estimation, bifurcation, asymmetry, bootstrapping, HIV prevention

## Abstract

The cusp catastrophe model is an innovative approach for investigating a phenomenon that consists of both continuous and discrete changes in one modeling framework. However, its application to empirical health and behavior data has been hindered by the complexity in data-model fit. In this study, we reported our work in the development of a new modeling method—cusp catastrophe regression (RegCusp in short) by casting the cusp catastrophe into a statistical regression. With the RegCusp approach, unbiased model parameters can be estimated with the maximum likelihood estimation method. To validate the RegCusp method, a series of simulations were conducted to demonstrate the unbiasedness of parameter estimation. Since the estimated residual variance with the Fisher information matrix method was over-dispersed, a bootstrap re-sampling procedure was developed and used as a remedy. We also demonstrate the practical applicability of the RegCusp with empirical data from an NIH-funded project to evaluate an HIV prevention intervention program to educate adolescents in the Bahamas for condom use. Study findings indicated that the model parameters estimated with RegCusp were practically more meaningful than those estimated with comparable methods, especially the estimated cusp point.

## 1. Introduction

Most statistical models for investigating medical, health, and behavioral outcomes are based on an assumption of linear and continuous change. But in the real world, changes in many outcomes are rarely linear and continuous as implemented in the corresponding statistical models. Contextual constrains can easily make a relationship between an influential factor and an outcome variable nonlinear and complex. In this case, a small change in an influential factor may result in abrupt and sudden changes in a disease, health, and behavioral outcome. In these situations, the application of a linear and continuous model will seriously limit the predictability of an influential factor on an outcome [1]. To deal with the nonlinearity and discrete characteristics, researchers extend the classical linear regression to include nonparametric kernel regression or regression with smoothing splines, additive models, random forests, support vector machine, to name a few [2,3]. Despite many advantages, these methods cannot be used to identify and incorporate medical, health, and behaviors that consist of multi-modes and sudden discrete changes under special conditions. The cusp catastrophe model is one that is capable of quantifying such a quantum mechanism to simultaneously consider linear and nonlinear relationships along with sudden jumps in outcome measures, as developed in Zeeman [4]. Catastrophe theory was initially developed in the 1970s [5] and subsequently used as a model in investigating accidents [6], adolescent alcohol and substance use [7,8], binge drinking [9], sexual initiation [10,11], nursing turnover [12], HIV prevention [10,13,14], therapy and program evaluation [15], and other health outcomes [16].

Computationally, two approaches have been established to implement the cusp catastrophe model in practice for data analysis. One is Guastello’s polynomial regression for cusp modeling analysis [6,15]. This method was developed for use in studies in which sampled participants or agents are measured longitudinally at two points in time (such as, before and after an intervention). The second method uses a stochastic differential equation as proposed by Cobb and his colleagues [17]. An R package “cusp” by Grasman et al. [18] was developed to implement this method. This paper explores a third approach by casting the cusp catastrophe into a statistical regression framework. The maximum likelihood method was thus used to minimize the difference between the observed outcome and the cusp catastrophe model predicted outcome as the true value. With this approach, the parameters of a cusp catastrophe model can be estimated using empirical data and the classical likelihood-based statistical inference.

## 2. An Overview of the Cusp Catastrophe Model

### 2.1. Cusp Catastrophe Model

The catastrophe model was proposed in the 1970 by Thom, a topological scientist [5]. The cusp model was subsequently popularized by several researchers [5,17,19,20,21]. Among seven elementary catastrophes, the cusp catastrophe gained particular population. Based on findings from published studies, the cusp catastrophe is highly relevant for researchers as both a guidance and methodology to investigate phenomena with bimodal distribution and sudden jumps, an inaccessible middle region between these two modes, a delay between these transitions, and deviation from a linear relationship between the response outcome measure and the predictors.

To implement the theory for real data analysis, the cusp catastrophe model is treated as a dynamic change system over time:

(1) $$\frac{dY}{dt}=\frac{dV\left(Y;\alpha ,\beta \right)}{dY}$$

where *Y* is the underlying true response or outcome variable, *α* is the asymmetry or normal control factor, and *β* is the bifurcation or splitting control factor. *V* is the potential function, which is defined as

(2) $$V\left(Y;\alpha ,\beta \right)=\alpha Y+\frac{1}{2}\beta Y^{2}-\;\frac{1}{4}Y^{4}$$

**Figure 1:**
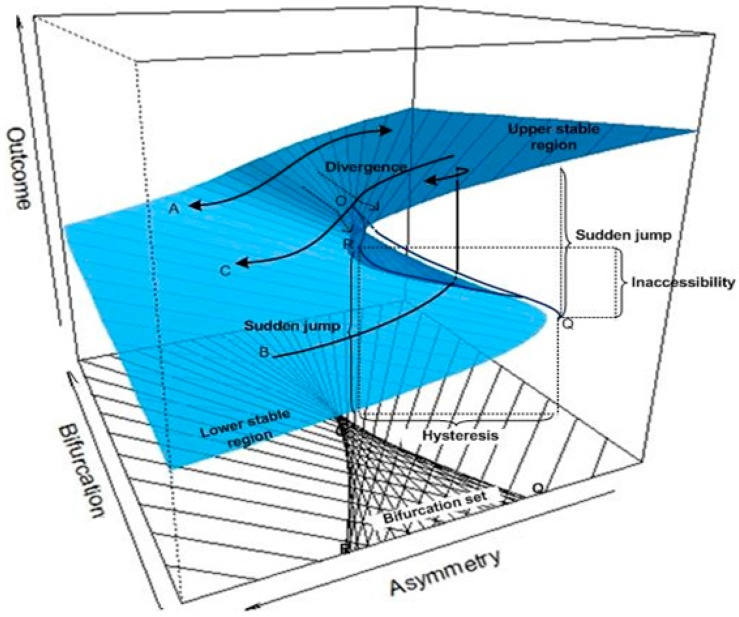
Cusp catastrophe model showing continuous and discrete changes in outcome (*Y*) on the equilibrium plane as the asymmetry variable α and the bifurcation variable β changes.

In Equation (1), if the right side moves to zero over time, changes in *Y* will also move to zero, which is defined as equilibrium plane. The equilibrium surface for a cusp model is depicted in Figure 1, showing changes in *Y* along with changes in in *α* and *β*.

It can be seen from Figure 1 that the variations in *Y* consist of two stable regions: the lower stable region located on the front left and the upper stable regions located on the front right. Beyond the two stable regions, *Y* is sensitive to changes in the two control variables, *α* and *β*. The outcome variable *Y* will become very unstable when it reaches the cusp region (the projected triangle-like region on the control plane (*α*, *β*). The cusp region is characterized by two lines with line OQ as the ascending threshold and line OR as the descending threshold. Within the cusp region, *Y* becomes extremely unstable. It can jump from the lower stable region to the upper stable region even with a very small change in *α* and/or *β.*

Paths A–C are three typical moving traces of *Y* on the cusp equilibrium plane. Path A shows a continuous and smooth relationship between *Y* and *α*. This type of relationship holds when the bifurcation variable *β* < O. Path B shows a discrete and sudden up-jumping relationship between *Y* and *α*. This type of relationship can happen when *β* > O. In this case, *Y* will suddenly jump from the low stable region to the upper stable region when *α* moves leftwards and passes line OQ. Likewise, Path C shows a discrete sudden dropping relationship between *Y* and *α.* This type of relationship holds when *β* > O. In this case, as *α* moves leftward and passes line OR, *Y* drops suddenly from the upper stable region to the lower stable region.

In empirical research, the cusp catastrophe model can also be used as a qualitative approach to describe nonlinear and complex relationships between predictors and outcome variables, including medical, health and behavioral outcomes. When using it qualitatively, researchers often focus on the identification of five catastrophe elements, known as the cusp catastrophe flags, as outlined by Gilmore [22]. These five flags can also be used in guiding quantitative cusp catastrophe modeling analysis.

### 2.2. Implementation of Cusp Catastrophe Model

With the cusp catastrophe model defined by Equation (1), Guastello successfully reframed the differential Equation (1) into a difference equation system [6,15] using a polynomial regression approach. The polynomial cusp regression method has been widely used in research to analyze health and behavioral data due to its high feasibility in implementation with SAS, SPSS, STATA, and R, or any software package with a linear regression capacity.

A second well-known cusp catastrophe modeling approach is the stochastic differential equation method (named thereafter as “SDECusp”) by Grasman and colleagues [18]. This modeling method can be implemented in R with a “cusp” package. In the SDECusp, cusp model, Equation (1) is reframed with a stochastic Wiener process to incorporate measurement errors into the observed outcome (denoted by y to be distinguished to the underlying true *Y*) by Cobb and his colleagues [17,20,21]. Mathematically, the SDECusp can be considered as an approach that casts the cusp catastrophe model in Equation (1) into a stochastic differential equation framework:

(3) $$dy=\;\frac{\partial V\left(y,\alpha ,\beta \right)}{\partial y}dt+dW\left(t\right)$$

where *dW* (*t*) is a white noise Wiener process with variance *σ^2^*.

Generally, the stochastic cusp model Equation (2) cannot be solved analytically. However, at the equilibrium state as time (*t*) approaches the infinity, we can obtain the probability density function (PDF) of the corresponding limiting stationary stochastic processes of *y* [7,23] as follows:

(4) $$f\left(y\right)=\frac{\Psi }{\sigma^{2}}exp\left[\frac{\alpha \left(y-\lambda \right)+\frac{1}{2}\beta {\left(y-\lambda \right)}^{2}-\frac{1}{4}{\left(y-\lambda \right)}^{4}\;}{\sigma^{2}}\right]$$

where ψ is a normalizing constant and λ is location parameter of the origin of *y*.

Using this PDF, the two control factors, α and β, are modeled as a linear regression to the predictors as proposed by Cobb [17,20,21] and Olive [24]. This PDF can be used with the statistical theory of maximum likelihood estimation to obtain model parameters along with the standard statistical inference, as demonstrated in the *R* Package “cusp” [18]. The SDECusp implemented with the R package is well suited for analyzing cross-sectional data, as indicated in reported studies by us [1,10,11,14,16] and others [25].

For diagnostics and comparisons to alternative linear regression and logistic regression models, Grasman [18] suggested the following four criteria:

(1) Negative log-likelihood values and the associated likelihood-ratio (LR) $\chi^{2}$-test where the smaller negative log-likelihood values, the better cusp model than the alternative models. If the LR $\chi^{2}$-test is used, the *p*-value < 0.05 would indicate a better fit of the cusp model.
(2) *R*^2^ defined as *R*^2^ = 1 − (error variance/variance of *y*) where the larger the *R*^2^, the better fit of the cusp model. Note that this is a pseudo-*R*^2^ since real *R*^2^ cannot be calculated for a cusp model.
(3) Akaike information criterion (*AIC*) [26] and Bayesian information criterion (*BIC*) [27] with a smaller *AIC* and/or *BIC* indicating better model fit.
(4) At least 10% (*α*, *β*) of the data pairs are located within the cusp region [28,29,30], or a nonlinear least squares regression with the logistic curve proposed by Hartelman [29], Hartelman et al. [31], and van der Maas and colleagues [32]:
  (5) $$y_{i}=\frac{1}{1+e^{-\alpha_{i/\beta_{i}^{2}}}}+\varepsilon_{i}$$

## 3. Cusp Catastrophe Regression

### 3.1. Introduction to the Cusp Catastrophe Regression Model

The cusp catastrophe model we developed is based on the regression principle to model an observed outcome to its associated true values, allowing for measurement errors. We thus named it as the Cusp Catastrophe Regression or RegCusp in short. Guided by statistical theories for regression modeling analysis, RegCusp is developed as a conceptual framework, and this approach is designated for analyzing continuous data. Following the cusp catastrophe model of Equation (1), we formulate the RegCusp model as follows:

(6) $$y_{i}=Y_{i}+\varepsilon_{i}$$

where $y_{i}$ (*i* = 1,…,*n*) are the observed outcome values, which are equal to the true *Y*_i_, plus measurement errors characterized by $\varepsilon_{i}$. The $\varepsilon_{i}$ are assumed to be normally distributed as $\epsilon_{i}\sim N\left(0,\;\sigma^{2}\right)$. The true *Y*_i_ in Equation (6) is then one of the real roots from the cusp catastrophe Equation (1) under equilibrium:

(7) $$\frac{dY_{i}}{dt}=\frac{dV\left(Y_{i};\;\alpha ,\beta \right)}{dY_{i}}=\alpha_{i}+\beta_{i}Y_{i}-Y_{i}^{3}=0$$

where $\alpha_{i}$ and $\beta_{i}$ are two control variables.

With the formulation of Equations (6) and (7), changes in *Y_i_* are converted from a canonical deterministic to a stochastic and probabilistic process. Consequently, sudden shifts in *Y_i_* are no longer restricted to the edge of the two threshold lines, but occur more frequently as *Y_i_* approaches toward the thresholds. Likewise, the stable regions based on the deterministic concept are also relaxed to allow for measurements of frequency or likelihood. In another word, a stable region means that *Y_i_* for any subject *i* has a large probability to be located in that area on the equilibrium plane of the cusp model.

### 3.2. Implementation Strategy

With observed data of *p* independent variables $\left(x_{1},\;\ldots ,x_{p}\right)$, the two control variables $\alpha_{i}$, and $\beta_{i}$, each are modeled using a linear combination of these variables as follows:

(8) $$\alpha_{i}=a_{0}+a_{1}x_{1}+\ldots +a_{p}x_{p}$$

(9) $$\beta_{i}=b_{0}+b_{1}x_{1}+\ldots +b_{p}x_{p}$$

All of the *p* independent variables $\left(x_{1},\;\ldots ,x_{p}\right)$ are used to composite both $\alpha_{i}$ and $\beta_{i}$. Any *x* with the model coefficient significantly different from zero in Equation (8) is used as the evidence supporting its role as an asymmetrical factor. Likewise, any *x* with a significant model coefficient in Equation (9) will indicate its function as a bifurcation factor. If the model coefficients of a variable *x* in both Equations (8) and (9) are significant, this variable possesses both asymmetry and bifurcation functions.

With the formulations above, the RegCusp is to estimate the model parameters $a=\left(a_{0},a_{1},\ldots ,a_{p}\right)$ and $b=\left(b_{0},b_{1},\ldots ,b_{p}\right)$ using Equations (8) and (9). The variance $\sigma^{2}$ of residuals can be estimated using Equation (6) to minimize the objective function of the sum of squared errors (SSE). The theoretical formulation is as follows:

(10) $$SSE(a,b|data)=\;\overset{n}{\underset{i=1}{\sum }}{\left(y_{i}-Y_{i}\right)}^{2}$$

This is equivalent to the maximum likelihood estimation with the following likelihood function:

(11) $$L\left(a,b,\sigma^{2}|data\right)=\;{\left(\frac{1}{\sqrt{2\pi \sigma }}\right)}^{n}exp\left(-\frac{\sum_{i=1}^{n}{\left(y_{i}-Y_{i}\right)}^{2}}{2\sigma^{2}}\right)$$

We implemented the RegCusp using Equation (11), taking advantages of the statistical properties of maximum likelihood estimation. With the likelihood function Equation (11), the theory of likelihood estimation was applied for parameter estimation and the associated statistical inferences on parameter significance and model selection. We implemented this estimation approach in *R*. The *R* function “optim” was used with the default Nelder–Mead algorithms to estimate the parameters and the gradients for standard errors.

It is worth pointing out that the likelihood function Equation (11) we defined for RegCusp differs in principle from that defined based on PDF in Equation (4) for SDECusp [17,18]. First, the log-likelihood function used by Grasman for SDECusp in the ‘cusp’ package is: $logL\left(a,b,\;w\right)=\;\overset{n}{\underset{i=1}{\sum }}log\left(\Psi_{i}\right)-\overset{n}{\underset{i=1}{\sum }}\left[\alpha_{i}y_{i}+\frac{1}{2}\beta_{i}y_{i}^{2}-\frac{1}{4}y_{i}^{4}\right]$. Therefore, SDECusp approach is based on the equilibrium limiting density for parameter estimation. In this case, data must be centered for analysis to estimate the model parameters. Second, in the SDECusp model, the observed outcome variable, $y_{i}$, is modeled as a linear combination to reflect the true value as $Y_{i}=w_{0}+w_{1}y_{i}$. Here, an extra set of parameters, ws, are introduced to the constructed cusp model. However, in our RegCusp model, data for the same observed variable $y_{i}$ is directly modeled, following the principles of regression analysis. In another word, our approach to solving the model is based on the comparison of observed data with cusp-model generated true values.

### 3.3. Special Properties of the Cusp Model

Theoretically, the RegCusp model in Equations (6)–(9) and parameters estimated using Equations (10) and (11) would hold the statistical properties of unbiasedness and efficiency as suggested by the maximum likelihood theory. However, as seen in Section 3.1, RegCusp is not a classical statistical model in which each combination of independent variables can produce one and only one outcome value. A RegCusp could have one, two, or three roots for each point ($\alpha_{i}$ and $\beta_{i}$) depending on its locations on the control plane. The number of roots of RegCusp is determined by the Cardan discriminant:

(12) $$\Delta \;=27\alpha^{2}-4\beta^{3}$$

From the Cardan discriminant Equation (12), it can be demonstrated that Equation (6) has one real root when Δ>0, and three real roots when Δ≤0. More specifically,

(a) if α=β=Δ=0, all the three roots would be the same corresponding to the *cusp point* (labeled O in Figure 1);
(b) if Δ=0, but α≠0 or β≠ 0, two of the three roots are equal which are corresponding to the two threshold lines OQ and OR in Figure 1 to characterize the boundary of the cusp region; and
(c) if Δ<0, and α≠0 or β≠ 0, all three roots are different which form the cusp region between OQ and OR in Figure 1.

Because of Equation (12) and the three different characteristics for a point (α, β) on the control plane, the RegCusp model is nonlinear and quantum consisting of both continuous and discrete process, beyond the traditional domain of mathematical and statistical modeling. Therefore, special investigations are used to warrant the correct selection of the right root based on Equation (7) to model Equation (6). We selected two traditionally developed modeling conventions, *delay convention* and *Maxwell convention* in this study to fulfill the root selection:

(1) The delay convention is to select the root from the $\frac{dV\left(Y;\alpha ,\beta \right)}{dY}=0$ in Equation (1) that are close to the observed *y*;
(2) The Maxwell convention is to select the root from the $\frac{dV\left(Y;\alpha ,\beta \right)}{dY}=0$ in Equation (1) that corresponds to the minimum of $V\left(Y;\alpha ,\beta \right)=\alpha Y+\frac{1}{2}\beta Y^{2}-\;\frac{1}{4}Y^{4}$.

### 3.4. Monte-Carlo Simulations

To validate the RegCusp modeling method, we tested it by a series of simulated data with known parameters. We found that the parameter estimates are unbiased with overestimated SEs. This over-dispersion effect is not uncommon in statistical modelling, such as in logistic regression and Poisson regression. To overcome the dispersion issue, we established a bootstrapping resampling method and used as a remedy. We report one scenario in this paper as an illustration.

In this scenario, data are simulated using Equation (6) with σ = 0.5 and *n* = 300. Two predictors of *x*_1_ and *x*_2_ are generated from the standard normal distribution with true regression parameter vectors ***a*** = (2, 2, 0), ***b*** = (2, 0, 2) using Equation (8) with *a*_2_ = 0 (implying that *x*_1_ is an asymmetry variable) and Equation (9) with *b*_1_ = 0 (implying that *x*_2_ is a bifurcation variable). This parameterization is used to assess if the RegCusp can correctly identify the role of the two variables while detecting the cusp catastrophe from the simulated data.

Data are generated with 5 steps:

- Step 1: randomly simulate *n* = 300 observations for *x*_1_ and *x*_2_ from the standard normal distribution and for the error term of $\varepsilon_{i}$ from normal distribution with mean 0 and standard deviation σ = 0.5;
- Step 2: calculate $\alpha_{i}$ and $\beta_{i}$ with Equations (8) and (9) using ***a*** = (2, 2, 0) and ***b*** = (2, 0, 2) as well as the *x*_1_ and *x*_2_ from Step 1;
- Step 3: for each set of $\alpha_{i}$ and $\beta_{i}$ from Step 2, we solve Equation (7) to obtain *Y_i_*. Based on the discussions in Section 3.2, we select one root corresponding to the Maxwell convention which is to minimize V (*Y_i_*, $\alpha_{i}$, $\beta_{i}$);
- Step 4: using *Y_i_* from Step 3 and $\varepsilon_{i}$ from Step 1, calculate *y_i_* based on the Equation (6);
- Step 5: with the data from Steps 1 to 4, estimate ***a*** and ***b*** using the maximum likelihood estimation in Equation (11).

The 5-step simulation procedure is repeated 5000 times and the results are summarized in Table 1. In the table columns named as “Mean” and “Med” represent the mean and median of the 5000 simulations. It is obvious that that the RegCsup can correctly identify model parameters since the parameter estimates are very close to the true values of ***a*** = (2, 2, 0) and ***b*** = (2, 0, 2). The simulation results suggest the unbiasedness from RegCusp.

**Figure 2:**
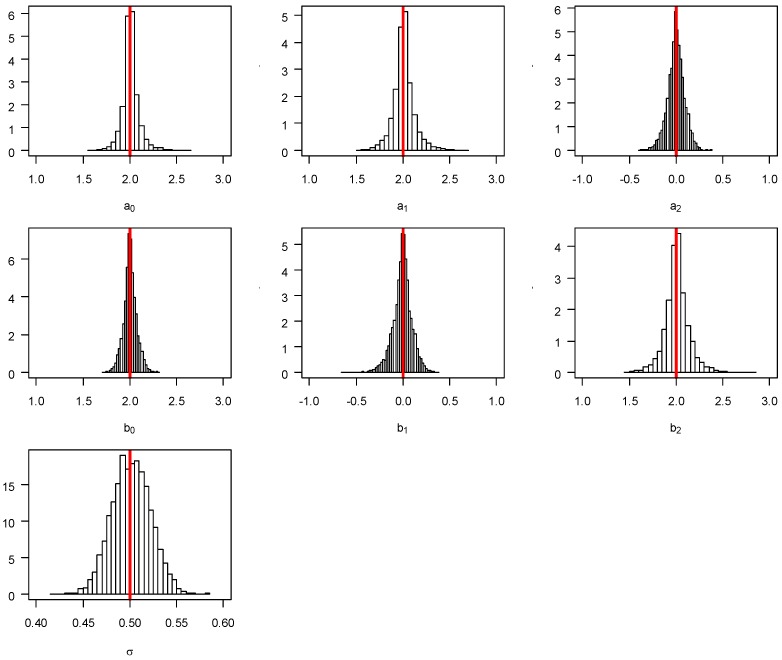
Sampling distributions of 5000 simulations for the parameters in RegCusp model. The *x*-axis denotes the range of the simulated parameters and *y*-axis denote the range of the associated probability density.

The unbiased property of the RegCsup model is also true for the estimated $\sigma^{2}$ generated using Equation (6) (mean = median = 0.51 from the 5000 simulations, not presented in the table). The unbiasedness of the point estimates of the RegCusp model is graphically evident in Figure 2 where the first six charts are for the estimated regression parameters and the last charts for $\sigma^{2}$.

In Table 1, the estimated sampling variance (column “EmpV”) and the average of estimated variance (column “EstV”) for the parameters of ***a*** and ***b*** were calculated from the 5000 simulations using the gradient Hessian matrix from the likelihood function. The empirical coverage probability (column “ECP”) was also presented in the table. Statistically, the sampling variance (i.e., “EmpV”) should be a consistent estimate of the true variance of the parameters and the estimated variance (i.e., “EstV”) should be close to “EmpV” so that the “ECP” should be close to 95%. A close assessment of the results in Table 1 reveals that the estimates of ECP are less than 95% for all of the parameters, indicating the lack of efficiency of the Hessian matrix for variance estimation. As shown in Figure 2, the distribution of the estimated parameters ***a*** and ***b*** from the 5000 simulations are highly leptokurtic which indicates that the variance estimation in RegCusp is inadequate and should be re-calibrated. To solve this problem, we resorted to a bootstrapping procedure.

### 3.5. Boostrapping Re-Sampling for Variance Estimation

Bootstrapping is a resampling procedure used in statistics to estimate variance when the typical Hessian matrix is inadequate. In any typical application for variance estimation, a small number of bootstrap in the range of 200 to 300 are sufficient and we used *B* = 300, which we found not efficient enough, so we choose *B* = 500 for illustration.

To correctly estimate the variance using the bootstrap procedure, the 5-step RegCusp simulation in Section 3.4 is now extended to include the sixth step of bootstrapping for a 6-step procedure as follows:

- Step 1 to Step 5, the same as in Section 3.4;
- Step 6: bootstrap the data from Step 4 and re-run the estimation Step 5 for *B* = 500 times to generate a bootstrapping sample.

The bootstrapping sample of size *B* = 500 can then be used for two purposes:

(1) to estimate the variances from these 500 samples. With the estimated bootstrapping variance, we can then construct the 95% CI for the parameter. Then the CIs are used to calculate the coverage probability (denoted by “ECP1”).
(2) to use the *B* = 500 samples to direct construct the 95% confidence intervals (CI) for each estimate and then the associated coverage probability (denoted by “ECP2”).

Bootstrapping results are summarized in Table 2. Column “EstV” is the estimated variance obtained from the 500 bootstrapping samples. These estimates are now much closer to the empirical variance “EmpV”. Column “ECP1” is the coverage probability using the bootstrapping variance from purpose (1), and the “ECP2” is the coverage probability using the bootstrapping CI from purpose (2). It can be seen from Table 2 that both coverage probabilities (i.e., ECP1 and ECP2) from the proposed bootstrapping method are much closer to 95% than the values obtained without bootstrapping in Table 1. Results in Table 2 indicate that our RegCusp model is valid with regard to both point and interval estimates.

## 4. Real Data Analysis

### 4.1. Data Source

To validate our new methods, surveys for an NIH funded project (Award #: R01 MH069229, PI: Stanton and Chen) were analyzed. The project was designed to test an educational HIV prevention program to encourage condom use among Bahamian adolescents [33]. The data were analyzed in a previous study to detect the intervention effect on the intention to use a condom with the SDECusp method [14]. In this study, we use data from the same source to test our RegCusp model in assessing factors other than intervention that may affect self-efficacy of condom use. Participants were public school students in Grade 9. They were randomly assigned to receive either intervention or control conditions. Data were collected in classroom settings using validated questionnaires. Follow-up data at Grade 12 (*n* = 1790, 40.6% male, mean age = 16.9 years, *SD* = 0.74) were analyzed.

Condom-use self-efficacy was used as the outcome variable *y* (mean score = 4.36, *SD* = 0.80). A total of six items were used to measure condom-use self-efficacy (Cronbach alpha = 0.81). A typical item read, “I could convince my partner that we should use a condom even if he (she) doesn’t want to.” Individual items were measured using a 5-point Likert scale (1 = *no*, *not at all*; 2 = *probably not*; 3 = *don’t know/unsure*; 4 = *probably yes*; and, 5 = *certainly yes*). Mean scores were computed over the six items. Participants who scored higher on the scale had higher self-efficacy to use a condom during sex.

HIV knowledge was used as the asymmetry variable *x*_1_ (mean = 14.29, *SD* = 2.39). Participants were asked 18 true/false questions regarding their knowledge on HIV transmission and prevention. An example question read, “A woman can get HIV if she has anal sex with a man who has HIV”. A participant received one point for each correct answer. Participants with higher total scores were considered more knowledgeable about HIV/AIDS. The score ranged from 0 (no HIV knowledge) to 18 (fully knowledgeable).

Response efficacy was used as the bifurcation variable *x*_2_ (mean score = 4.36, *SD* = 0.88). Response efficacy measures the perceived effectiveness of condom use in preventing HIV infection. This variable was measured using three items (Cronbach alpha = 0.80). An example item read: “Condoms are an important way to prevent you from getting a sexually transmitted disease (STD).” Items were assessed using the 5-point Likert scale with 1 (*strongly disagree*) and 5 (*strongly agree*).

According to behavioral theory and the cusp catastrophe model, there should be a positive relationship between HIV knowledge *x*_1_ and the outcome variable condom-use self-efficacy *y*. Different from conventional regression analysis, in cusp catastrophe model, the continuous relationship between *x*_1_ and *y* can be bifurcated by *x*_2_, the response efficacy or perceived effectiveness of condom use. When *x_2_* is located behind the bifurcation point O, the positive relationship between *x*_1_ and *y* will be continuous; however, when the bifurcation variable *x*_2_ is located in front of point O, changes in *y* will become discrete with two *y* values distributed at all (*x*_1_, *x*_2_) points corresponding to the cusp equilibrium surface.

### 4.2. Linear Regression Analysis

Results from linear regression analysis as a conventional approach are presented in Table 3. Results in the table indicate a significant and positive association of *x*_1_ (HIV knowledge, β = 0.0080, *p* < 0.01) and *x*_2_ (the perceived condom efficacy, β = 0.2033, *p* < 0.01) with *y* (condom-use self-efficacy). The positive relationships are supported by the behavioral theory. However, the *R*^2^ estimated from the linear regression, including the adjusted *R*^2^ was less than 8%, rather small.

### 4.3. SDECusp Analysis

The data analyzed using the linear regression method in the previous section were re-analyzed using the SDECusp. The analysis was implemented using Grasman’s R package “cusp” [18]. The package is available free of change and detailed description of this “cusp” package is also available at http://cran.r-project.org/web/packages/cusp/vignettes/Cusp-JSS.pdf.

Before analysis, the two predictor variable and the outcome variable were standardized, as suggested by the R package as denoted by A and B in Table 4 below, which summarizes the main findings from the SDECusp modeling analysis.

First, the SDECusp results indicate that the asymmetry variable *x*_1_ (HIV knowledge, *a* = 0.1760, *p* < 0.01) and the bifurcation variable *x*_2_ (perceived condom efficacy, *b* = 0.2147, *p* < 0.01) were highly significant in predicting the outcome variable *y* (condom use self-efficacy), as observed from the linear regression analysis.

Furthermore, the estimated *R*^2^ for the cusp modeling was 34%, much greater than 7.8%, the *R*^2^ for linear regression model. This result indicates the superiority of cusp catastrophe model over linear regression model in quantifying the relationship between the predictor and the outcome variable. Chi-square test also demonstrated that the data could be better modeled with a cusp catastrophe than a linear regression model (*χ*^2^ = 1110, *df* = 2, *p* < 0.0001).

A more in-depth review of the modeling results indicated that differences between the estimated coefficient *a*_1_ for the asymmetry variable and *b*_1_ for the bifurcation variable were much smaller from cusp catastrophe model (0.1760 and 0.2147) than from the linear regression model (0.0080 and 0.2033).

**Figure 3:**
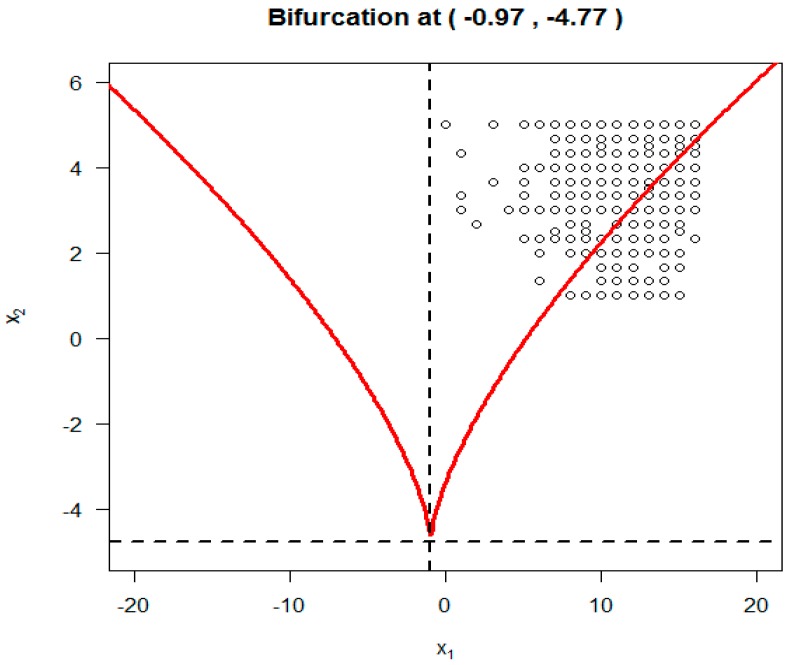
Estimated bifurcation point from the SDECusp modeling.

To display the SDECusp model fitting over the cusp catastrophe control plane (α = asymmetry, β = bifurcation), as seen in Figure 1 (i.e., the 2-d contour plane projected from cusp 3-d surface), we make use of the parameter estimates (i.e., *a*_0_, *a*_1_, *b*_0_ and *b*_1_) from Table 4 and the standardized A and B to calculate the standardized asymmetry control factor α and the bifurcation control factor β. These standardized factors are then transformed back to the original scales as seen in Figure 3. All of the data points (*n* = 1790) are plotted in Figure 3.

Overall, 1723 out of the 1790 were located within the cusp region. If this is true, that condom-use self-efficacy among 96% (1723/1790) of the participants was not stable but subject to rapid change. The remaining 272 data points were located in the upper stable region; they present the participants with high condom-use self-efficacy. No data point was observed anywhere else on the equilibrium plane.

### 4.4. Analysis with RegCusp Modeling Method

We analyzed the same data using the RegCusp method. With the RegCusp, the estimated residual variance was 0.982. The estimated two parameters for the asymmetry were *a*_0_ = −0.083, *a*_1_ = 0.094 (*p* < 0.01 for both). Likewise, the estimated two parameters for the bifurcation variable were *b*_0_ = 1.568; *b*_1_ = 0.672 (*p* < 0.01).

From RegCusp fitting, the control variables, *x*_1_ (HIV knowledge) and *x*_2_ (response efficacy) both significantly and positively predicted the outcome variable *y* (condom use self-efficacy). The results were consistent with those obtained from linear regression (Section 4.2) as well as the SDECusp modeling analysis (Section 4.3) with regard to the prediction direction. Furthermore, the estimated coefficients for the two control variables in RegCusp were closer to each other as compared to those estimated with the SDECusp modeling method.

**Figure 4:**
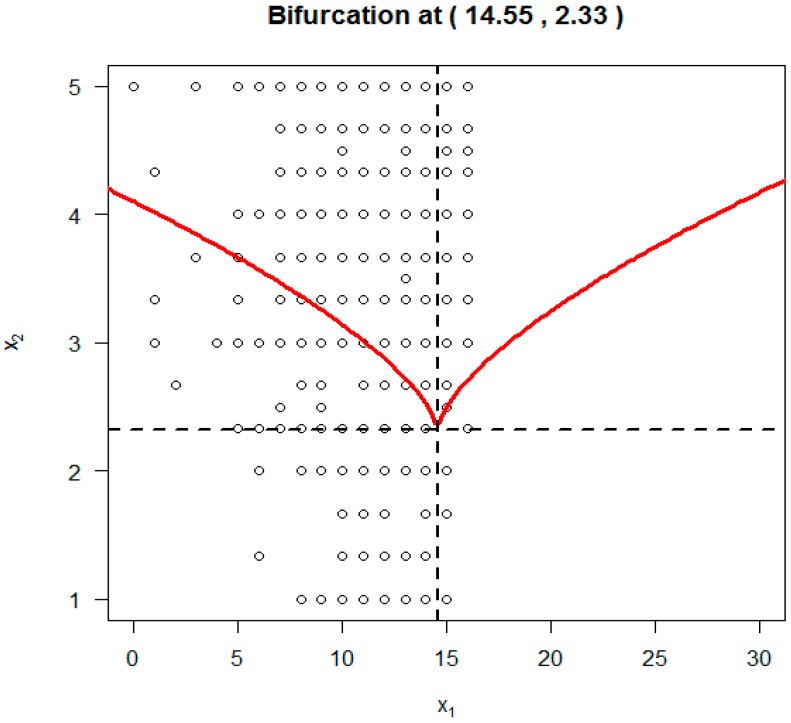
Cusp region from RegCusp model.

As another comparison to SDECsup, Figure 4 depicts the cusp point O (α, β) using results from the RegCusp with *x*_1_ (HIV knowledge) = 14.55, and *x*_2_ (response efficacy) = 2.33. It is clear that these two values are completely within the range of the original data for the two predictor variables. Furthermore, the two values of the cusp point O were practically meaningful. According to the estimated cusp point, to have a sudden change in condom-use self-efficacy, a student should have adequate knowledge with s knowledge score of at least 14.55 on the 18-item HIV Knowledge Scale. To achieve a sudden change, a student should have a minimum score of 2.33 on the Response Efficacy measure within the original score range of 1 (*strongly disagree*) and 5 (*strongly agree*). Therefore, when compared to the results from SDECusp/R “cusp” package, the results from the RegCusp was more convincing.

Results of the estimated cusp point using the RegCusp modeling suggest that when the perceived effectiveness of condom for HIV prevention was <2.33 (as seen in Figure 4) on the response efficacy scale, the outcome variable will change gradually and smoothly along with changes in HIV knowledge. Students who have gained more HIV knowledge will be more likely to have strong self-efficacy in correctly using a condom during sex.

However, when the perceived effectiveness of condom for HIV prevention was >2.33 on the response efficacy scale, changes in HIV knowledge may lead to very different results: (a) For students without much confidence in using a condom during sex, a sudden increase is likely in their perceived ability to use condoms likely; (b) For students with lots of self-confidence, a sudden drop is also likely for their perceived ability to use condoms; and, (c) the point when a sudden change occurs is controlled by the two threshold lines marked in red. As an example, for students with HIV knowledge <14.55, changes in the response efficacy for condom use between 2.3 (*somewhat ineffective*) and 4.0 (*somewhat effective*) or 5.0 (*very effective*) can, depending on knowledge level, trigger a sudden jump in self-confidence fort condom use during sex.

## 5. Discussion

Cusp catastrophe model is theory-grounded with promising application in analyzing empirical data. Evidence from diverse resources including findings from this study support the notion that cusp catastrophe model is a promising analytical tool. It will help researchers to expand their research horizons and to investigate nonlinear and quantum changes beyond the conventional Euclidean space [1]. The cusp catastrophe model has been applied successfully in different scientific fields. However, its application in the social, behavioral, medical and public health sciences is criticized. For more detailed discussion, please see [34,35].

In the past decades, a number of researchers have attempted different methods to turn the deterministic Cusp catastrophe model into a statistical model to promote its application in data analysis. Their efforts provide useful insight and large amount of data. Typical achievements include the development of the polynomial cusp regression approach, following the polynomial regression method, as discussed by Guastello and colleagues [6,15,36] and the establishment of the stochastic cusp model, capitalizing on the maximum likelihood estimation theory, as discussed by Cobb [17,20,21] and Grasman and others [18]. Despite certain limitations [4,13,14], these methods make it possible for researchers to examine issues with nonlinear characteristics that are very common but ignored in social, behavioral, medical and health sciences.

Along with the methodology advancement, we reported the RegCusp modeling method from our own research. Deeply rooted in the previous research, this novel RegCusp modeling method adds another tool to fit cusp catastrophe models with really data. We have demonstrated and proofed the unbiasedness and efficiency of the RegCusp in parameter estimation through simulation studies and empirical data analyses. We also innovatively used a bootstrapping procedure to resolve variance estimation issue, recognizing that the Fisher information matrix is limited in estimating the residual variance for cusp catastrophe modeling.

## 6. Conclusions

Based on findings from this study, we conclude that the RegCusp advanced methodology research with regard to the cusp catastrophe modeling and will promote the application of the cusp catastrophe model in research. Several strengths of our RegCusp modeling method are summarized below:

(1) The RegCusp modeling method is statistical theory-grounded. We established the RegCusp method guided by the nontraditional, nonlinear regression principle and capitalized on the maximum likelihood estimation theory. We are the first to attempt to convert the deterministic cusp catastrophe model into a statistical model following the regression analysis principles. The success of our work suggests great potentials to solve the cusp catastrophe as well as other catastrophe models.

(2) The unbiasedness and efficiency of the RegCusp are supported with intensive simulation studies. The validity of the RegCusp method has been subject to extensive simulation tests. Results from simulations studies indicate that the RegCusp has achieved both the unbiased parameter estimation and efficient variance estimation.

(3) Verified with empirical data: By analyzing empirical data, we further show that the estimated model parameters with the RegCusp method, particularly the cusp point O and threshold lines are practically meaningful than those estimated with other methods.

(4) Obtaining information regarding the threshold lines is of great significance for the use of the cusp catastrophe model in research. Unbiased estimation of the two threshold lines for a cusp model requires an unbiased estimation of the cusp point for bifurcation. In all of the published methodology studies, no one reported results on cusp point and threshold lines when using cusp models to analyze empirical data. We are the first to report such results with empirical data. By investigating and comparing different methods with real data, we demonstrate the advantage of our RegCusp approach in solving the problem over the published methods.

Although findings from our studies are highly encouraging, additional studies are needed to further the RegCusp modeling method. Theoretically, we need to gain more insight into the statistical properties of RegCusp approach. Methodologically, it is unclear why the Fisher information matrix cannot obtain unbiased estimate residual variance for cusp catastrophe models. Additional research is needed to expand the RegCusp modeling method to analyze non-continuous variables, such as data with binary, polynomial, and Poisson distributions.

## Figures and Tables

**Table 1:** Result from simulation studies (5000 Simulations).

Parameter	Mean	Med	EmpV	EstV	ECP
***a*_0_**	2.0094	2.0035	0.0079	0.9525	0.3323
***a*_1_**	2.0106	2.0062	0.0134	1.2496	0.2558
***a*_2_**	−0.0014	−0.0009	0.0082	0.3232	0.2502
***b*_0_**	2.0038	2.0016	0.0048	0.3240	0.3093
***b*_1_**	−0.0069	−0.0029	0.0102	0.7649	0.2483
***b*_2_**	2.0115	2.0057	0.0169	1.4016	0.2246

**Table 2:** Results from Simulation Studies with Bootstrapping (*n* = 500).

Parameter	Mean	Med	EmpV	EstV	ECP1	ECP2
***a*_0_**	2.023	2.014	0.0370	0.0369	0.949	0.953
***a*_1_**	2.031	2.009	0.0614	0.0615	0.951	0.949
***a*_2_**	−0.014	0.002	0.0363	0.0365	0.952	0.950
***b*_0_**	2.005	2.009	0.0195	0.0194	0.948	0.951
***b*_1_**	−0.023	−0.009	0.0467	0.0466	0.949	0.953
***b*_2_**	2.027	2.010	0.0787	0.0786	0.949	0.948

**Table 3:** Results from linear regression analysis.

Parameter	Estimate	Standard Error	*t* Value	*p* Value
(Intercept)	2.877	0.119	24.245	<0.0001
*x*_1_	0.047	0.008	5.935	<0.0001
*x*_2_	0.203	0.020	10.065	<0.0001

Standard error of the residuals: 0.7677, *df* = 1992; multiple *R*^2^ = 0.07986, Adjusted *R*^2^ = 0.07894; F = 86.45 (2, 1992), *p* ≤ 0.01.

**Table 4:** Results from SDECusp modeling analysis.

Parameter	Estimate	Std. Error		*z* Value	Pr (>|t|)
A (Intercept, *a*_0_)	1.076	0.049		21.967	<0.0001
A (Slope, *a*_1_)	0.176	0.026		6.839	<0.0001
B (Intercept, *b*_0_)	2.243	0.082		27.332	<0.0001
B (Slope, *b*_1_)	0.215	0.035		6.073	<0.0001
Y (Intercept, *w*_0_)	1.359	0.021		64.199	<0.0001
Y (Slope, *w*_1_)	0.798	0.013		62.038	<0.0001
	**R.Squared**	**logLik npar**	**AIC**	**AICc**	**BIC**
Linear model	0.0798	−2747.254	5502.51	5502.53	5524.91
Cusp model	0.3381	−2192.024 6	4396.05	4396.09	4429.64

Chi-square test comparing linear regression model with cusp catastrophe model. X^2^ = 1110, *df* = 2, *p* < 0.000.
